# Multiple micromeningiomas coexist with primary lung adenocarcinoma: A case report

**DOI:** 10.1111/1759-7714.14872

**Published:** 2023-03-28

**Authors:** Jindong Chen, Ling You, Jingsi Dong

**Affiliations:** ^1^ Department of Lung Cancer Center, West China Hospital Sichuan University Chengdu China

**Keywords:** coexistence, lung cancer, pulmonary meningioma

## Abstract

Cases of multiple ectopic meningiomas in the lung coexisting with pulmonary malignancies are extremely rare in the clinic. On imaging, it is difficult to distinguish multiple ectopic meningiomas from lung cancer, which puts forward higher requirements for treatment. A 65‐year‐old female patient was admitted to our department for multiple nodules in both lungs. The patient underwent thoracoscopic wedge resection and segmental resection. Postoperative pathological examination found lung meningioma, atypical adenomatoid hyperplasia (AAH), carcinoma in situ (AIS), invasive adenocarcinoma, and other pathological types. In this case, pulmonary meningioma, AAH, AIS, and invasive adenocarcinoma of various pulmonary nodules were observed. This case, which has not been reported before, is unique in that it has multiple pathologic types in one organ. This puts forward higher requirements for clinical diagnosis and treatment.

## Graphical abstract

Multiple lung micromeningiomas, adenocarcinoma and benign nodules on the computed tomography scan.

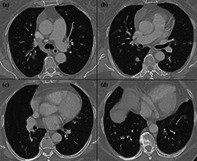

## INTRODUCTION

Meningioma is usually a benign disease of the central nervous system. Ectopic meningiomas account for a relatively small proportion of meningiomas, and ectopic meningiomas in lungs are rarer than in other organs.^1^ Cases of multiple ectopic meningiomas in lungs coexisting with pulmonary malignancies are extremely rare in the clinic. On imaging, it is difficult to distinguish multiple ectopic meningiomas from lung cancer, which puts forward higher requirements for treatment. In this article, we report a case of pulmonary meningiomas complicated with pulmonary malignancy.

## CASE PRESENTATION

**FIGURE 1:**
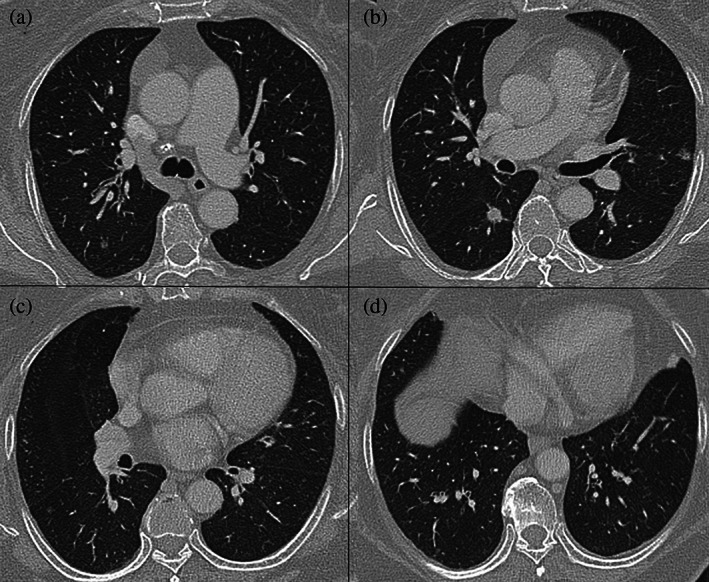
Multiple lung nodules on the CT scan

A 65‐year‐old woman underwent a computed tomography (CT) scan of her chest and detected multiple lung nodules during a routine health checkup. The patient has a history of hypertension, diabetes mellitus type 2, and resection of back lipomas. She denied history of tuberculosis, and she was a non‐smoker. She did not have any respiratory symptoms and the physical examination was all right. Chest CT revealed unequal nodules in both lungs, including 5 nodules in the left lung and 9 nodules in the right lung. These multiple nodules were initially suspected to be multi‐origin lung cancer (Figure 1). The patient underwent a series of tests, including head CT, abdominal CT, pelvic CT, fiber bronchoscopy, tuberculosis disease tests, and serum tumor markers (such as carbohydrate antigen 19–9 and carcinoembryonic antigen). These tests revealed no obvious abnormalities. For pathological diagnosis and treatment, video‐assisted thoracic wedge resection and segmental resection was performed on the posterior segment of the upper lobe of right lung, the lateral and posterior basal segments of lower lobe of the right lung, and the superior segment of lower lobe of the right lung at the same time. These resected nodules were examined pathologically (Table 1). Microscopically, two nodules (nodule 1 and 2) were found in the posterior segment of the upper lobe of the right lung, with a diameter of 2 mm. The histology was chronic inflammation of lung tissue, focal fibrous hyperplasia with carbon deposition, and focal alveolar epithelial hyperplasia. These two nodules were considered atypical adenomatous hyperplasia (AAH). Two nodules were found in the lateral basal segment of the lower lobe of the right lung. One (nodule 3) is a microscopic meningeal epithelioid nodule and the other (nodule 4) is a small alveolar epithelial atypical hyperplasia. In the posterior basal segment of the lower lobe of the right lung, a tiny meningeal epithelioid nodule (nodule 5) was found. There were 4 nodules in the superior segment of the lower lobe of the right lung. Among them, no. 1 (nodule 6) was infiltrating adenocarcinoma, which included both acinar type and adherent type. No. 2 (nodule 7) was a microscopic meningeal epithelioid nodule. No. 3 (nodule 8) was adenocarcinoma in situ (AIS). No. 4 (nodule 9) was atypical adenomatous hyperplasia (AAH). No tumor involvement was detected at the bronchial stump. Immunohistochemistry tests of these micromeningeal epitheliod nodules showed epithelial membrane antigen (EMA) (+), progesterone receptor (PR) (+), somatostatin receptor 2 (SSTR2) (+), CD31 (−), CK7 (−), thyroid transcription factor‐1 (TTF‐1) (−), CD68 (−), Ki‐67 (+, 3%), and S‐100 (−). The pathological diagnosis was minute pulmonary meningothelial‐like nodules (MPMN). Immunohistochemical examination of nodule 6, which was infiltrating adenocarcinoma, revealed CK7 (+), TTF‐1 (+), NapsinA(+), CK5/6 (−), ALK‐V(−), and ROS‐1 (−).

**TABLE 1:** Location, histology, and immunohistochemistry of multiple nodules

Nodule	Location	Histology	Immunohistochemistry
1 and 2	Posterior segment of the upper lobe of the right lung	Atypical adenomatous hyperplasia	–
3	Lateral basal segment of the lower lobe of the right lung	Meningeal epithelioid nodule	EMA(+), PR(+), SSTR2(+), CD31(−), CK7(−), TTF‐1(−), CD68(−), S‐100(−), Ki‐67 (+, 3%)
4		Atypical alveolar hyperplasia	–
5	Posterior basal segment of the lower lobe of the right lung	Meningeal epithelioid nodule	EMA(+), PR(+), SSTR2(+), CD31(−), CK7(−), TTF‐1(−), CD68(−), S‐100(−), Ki‐67 (+, 3%)
6	Superior segment of the lower lobe of the right lung	Infiltrating adenocarcinoma	CK7(+), TTF‐1(+), NapsinA(+), CK5/6(−), ALK‐V(−), ROS‐1 (−)
7		Meningeal epithelioid nodule	EMA(+), PR(+), SSTR2(+), CD31(−), CK7(−), TTF‐1(−), CD68(−), S‐100(−), Ki‐67 (+, 3%)
8		Adenocarcinoma in situ	–
9		Atypical adenomatous hyperplasia	–

## DISCUSSION

Pulmonary meningioma is rare among meningiomas.^2^, ^3^ It is usually discovered incidentally on a CT scan during a physical examination and there are usually no typical respiratory symptoms.^4^, ^5^ Pulmonary meningiomas are often distributed under the pleura, of ground‐glass opacity, and generally have no malignant signs such as protrusions and pleural indentations.^6^ Pulmonary meningiomas are imageologically similar to pulmonary ground glass nodules and have no distinctive imaging features. The superior segment of the lower lobe of the right lung in this patient had a large mixed density nodule and multiple ground glass nodules scattered elsewhere, which were often misdiagnosed as multiple lung carcinomas in situ or lung cancer metastases in clinical practice.

A rare primary pulmonary meningioma in a 59‐year‐old patient was first reported by Kemnitz et al.^7^ in 1982. Up to now, more than 40 cases of pulmonary meningioma have been reported in the literature. The co‐existence of primary lung cancer and pulmonary meningioma has not been reported before. In our case, the patient had scattered nodules of different sizes in both left and right lungs, and the benign nodules could not be distinguished from the malignant ones. Therefore, treatment plans can only be evaluated according to the patient's physical status, location of the lesion, patient's prognosis, etc. Because the patient had multiple nodules in both lungs, we were unable to remove all the suspicious lesions. According to the characteristic of slow growth of ground glass nodules, the right lung was treated with wedge resection combined with segmental resection, whereas the left lung was on observation of regular clinical follow ups.

In this case, pulmonary meningioma, AAH, AIS, and invasive adenocarcinoma of various pulmonary nodules were observed. This interesting phenomenon has given rise to some new problems, such as whether the co‐occurrence of these pathological types is accidental, whether there are certain correlations between them and whether these nodules evolve from one another in different microenvironments. These speculations need to be confirmed by a large number of subsequent studies.

In summary, this case, which has not been reported before, is unique in that it has multiple pathologic types in one. This puts forward higher requirements for clinical diagnosis and treatment.

## AUTHOR CONTRIBUTIONS

Conceptualization, Jingsi Dong; methodology, Jindong Chen; software, Jingsi Dong; validation, Jingsi Dong; writing—original draft preparation, Ling You; writing—review and editing, Jindong Chen; supervision, Jingsi Dong; funding acquisition, Jingsi Dong; Jindong Chen and Ling You contribute equally to this work. All authors have read and agreed to the published version of the manuscript.

## FUNDING INFORMATION

This work was supported by the Department of Science and Technology of Sichuan Province, Grant/Award Number: 2022YFS0219.

## CONFLICT OF INTEREST STATEMENT

The authors declare no conflflict of interest.
